# RPL22L1 is a novel biomarker for prognosis and immune infiltration in lung adenocarcinoma, promoting the growth and metastasis of LUAD cells by inhibiting the MDM2/P53 signaling pathway

**DOI:** 10.18632/aging.206096

**Published:** 2024-08-28

**Authors:** Shigui Xing, Dongbing Li, Qi Zhao

**Affiliations:** 1 Department of Thoracic Surgery, Nanjing Gaochun People’s Hospital, Nanjing 211300, Jiangsu, China; 2 Scientific Research Center, Beijing ChosenMed Clinical Laboratory Co., Ltd., Beijing 100176, China; 3 Department of Pulmonary and Critical Care Medicine, Nanjing Drum Tower Hospital, The Affiliated Hospital of Nanjing University Medical School, Nanjing 210008, Jiangsu, China

**Keywords:** RPL22L1, biomarker, prognosis, lung adenocarcinoma, immune infiltration, MDM2/P53

## Abstract

The ribosomal protein L22-like1 (RPL22L1) is a constituent of the 60 S ribosomal subunit whose function in lung adenocarcinoma (LUAD) remains ambiguous. This study aims to elucidate the role of RPL22L1 in LUAD through a thorough analysis and experimental validation. Our findings indicate that RPL22L1 exhibits abnormal expression patterns in various cancer types, including LUAD. Moreover, a statistically significant association was observed between elevated levels of RPL22L1 expression in LUAD patients and several clinical parameters, such as pathological stage (p = 0.0083) and gender (p = 0.0038). The high expression of RPL22L1 in LUAD demonstrated a significant association with poorer overall survival (OS) (p = 0.005), progression-free survival (PFS) (p = 0.027), and disease-specific survival (p = 0.015). The expression of RPL22L1 in LUAD (p = 0.005) was identified as an independent prognostic factor. Additionally, RPL22L1 expression in LUAD was found to be correlated with immune infiltration, immune checkpoint genes, TMB/MSI, and mRNAsi. Notably, the expression of RPL22L1 exhibited significant negative correlations with 1-BET-762, Trametinib, and WZ3105 in LUAD. The RPL22L1 gene exhibited up-regulation in multiple individual cells of LUAD, leading to a comparatively shorter PFS in the RPL22L1 variant group as opposed to the RPL22L1 variant-free group in LUAD. Significantly increased expression of RPL22L1 was noted in LUAD cell lines, where it was found to enhance the growth and metastasis of LUAD cells by suppressing the MDM2/P53 signaling pathway. Therefore, RPL22L1 may serve as a promising prognostic biomarker and therapeutic target for patients with LUAD.

## INTRODUCTION

Lung cancer exhibits a substantial prevalence and fatality rate, with lung adenocarcinoma (LUAD) accounting for approximately 40% of all cases worldwide. According to projections made by the American Cancer Society, approximately 127,070 individuals are expected to succumb to lung cancer by the end of the year 2023, representing a substantial 20% of the total mortality attributed to cancer [1]. Currently, considerable advances have been achieved in the domain of LUAD treatment, with a particular focus on the development of personalized therapeutic strategies. Notable progress in this field encompasses the widespread implementation of immune checkpoint inhibitor (ICI) therapy and targeted therapy [2]. The effectiveness of immunotherapy in treating locally advanced and metastatic non-small cell lung cancer is restricted to only 20% of patients, primarily due to the complex and diverse characteristics of the tumors [3]. Consequently, the prognosis for LUAD remains unfavorable, resulting in a five-year survival rate of around 18% [4]. Therefore, there is a pressing need to enhance both the prognosis and treatment of LUAD.

Ribosomal proteins, particularly the ribosomal protein L22-like1 (RPL22L1), play a critical role in the pathogenesis of cancer [5, 6]. RPL22L1, a component of the 60S subunit regulated by RPL22, has been found to be significantly upregulated in glioblastoma (GBM) and hepatocellular carcinoma (HCC), leading to a poorer outcome for patients [7, 8]. RPL22L1 demonstrates prognostic significance in kidney renal clear cell carcinoma (KIRC), emerges as a key gene in the progression of prostate cancer (PRAD), and may serve as a potential target for therapeutic interventions in PRAD [9–11]. RPL22L1 may function as a prognostic marker for colorectal cancer (CRC) and has the potential to serve as a prognostic indicator and/or therapeutic target for ovarian cancer (OC) [12, 13]. However, the role and underlying mechanisms of RPL22L1 in LUAD remain unclear.

Currently, the precise involvement of RPL22L1 in LUAD remains uncertain. This study seeks to analyze the expression of RPL22L1 in pan-cancer and LUAD, utilizing data from the Cancer Genome Atlas (TCGA) database to evaluate its diagnostic relevance [8]. The study explores the relationship between RPL22L1 expression levels and clinical features, as well as prognosis in LUAD. The investigation delves into potential regulatory networks linked to RPL22L1, including its association with tumor microenvironment (TME), tumor mutational burden (TMB)/microsatellite instability (MSI), mRNA stemness index (mRNAsi), and drug sensitivity in LUAD [14]. We explore RPL22L1 expression in LUAD single-cell sequencing and the genomic variation and clinical significance of RPL22L1 in LUAD. The expression of RPL22L1 was validated using GSE87340. Next, RPL22L1 was transfected into the LUAD cell line and demonstrated by Western blot. The effects and mechanisms of RPL22L1 on the proliferation of LUAD cell lines *in vitro* and *in vivo* were explored by the CCK8 assay, clone formation, wound healing, transwell, flow cytometry, and xenografting assay.

## MATERIALS AND METHODS

### Expression analysis of RPL22L1 in TCGA-LUAD in pan cancer and LUAD

A total of 539 patients diagnosed with LUAD and 59 normal tissue samples were obtained from TCGA [15, 16].

The RNAseq data in transcripts per million (TPM) format from TCGA (https://portal.gdc.cancer.gov) and GTEx were processed consistently using the Toil method and accessed via the UCSC XENA platform (https://xenabrowser.net/datapages/) [17]. Pan-cancer data from TCGA and normal tissue data from GTEx were extracted and processed using a log 2 (value + 1) transformation [15]. Visualization of the data was performed using the ggplot2 package, with statistical analysis conducted using the appropriate methods from the stats and car packages based on the data format characteristics [18].

The RNAseq data were retrieved and organized utilizing the STAR process of the TCGA-LUAD project, and subsequently converted into TPM format. The data were further processed by applying a log2 (value + 1) transformation.

### The relationship between RPL22L1 and clinical features and its diagnostic value

We use software version R (4.2.1) for the statistics. The data filtering strategy was to remove normal and remove nonclinical information. Clinical factors examined were T stage, N stage, pathological stage, gender, age, and anatomical neoplasm subdivision.

Data were ROC-analyzed using the pROC package and the results were graphically represented using ggplot2 [19].

### The relationship between RPL22L1 and the prognosis

Proportional risk hypothesis testing and fitted survival regressions were conducted utilizing the survival package, with the results visualized using the survminer and ggplot2 packages. Prognostic factors considered in the analysis included overall survival (OS), progression-free survival (PFS), and disease-specific survival (DSS) [18].

The forest plots were generated using the software R (version 3.6.3) and the ggplot2 package [20].

The nomogram plot was created utilizing the rms package and the survival package [21]. The prognostic type was OS. Variables include the T stage, pathological stage, tumor status, and RPL22L1.

### Gene set enrichment analysis (GSEA)

We use the software R (version 4.2.1) and the R package clusterProfiler [4.4.4]. Following the conversion of molecule IDs in the input data, GSEA was carried out using the clusterProfiler package. The study focused on human (Homo sapiens) species, with a set of reference genes identified as c2.cp.all.v2022.1.Hs.symbols.gmt [All Canonical Pathways] (3050) [22–24].

### The relationship between RPL22L1 and immune infiltration, immune score, and immune checkpoints

The immune infiltration of the cloud data was calculated based on the ssGSEA algorithm from the R package GSVA [1.46.0], using markers for 24 immune cells provided in reference [25].

The R (4.2.1) version and associated packages, including ggplot2 (3.3.6), stats (4.2.1), and car (3.1-0), were utilized for data processing in this study. Following the grouping of key variables, appropriate statistical methods from the stats and car packages were chosen based on the data format characteristics, with statistical analysis contingent upon meeting specific requirements. Data visualization was conducted using the ggplot2 package, and the statistical method employed was the Wilcoxon rank sum test. Additionally, matrix and immunity scores for cloud data were calculated using the R package-estimate [1.0.13] [26].

The RNAseq data (level 3) and corresponding clinical information of LUAD were obtained from TCGA. Transcripts associated with immune checkpoints, such as SIGLEC15, IDO1, CD274 (PD-L1), HAVCR2, PDCD1, CTLA4, LAG3, and PDCD1LG2, were identified [14, 27]. The expression values of the eight genes were extracted for the purpose of examining their association with immune checkpoint genes. Statistical analysis was conducted using R software version 4.0.3, with the rank sum test utilized to identify differences between the two data groups. A p-value of less than 0.05 was deemed statistically significant, unless specified otherwise.

### The relationship between RPL22L1 and TMB/MSI and cancer stem cells (CSCs)

TMB serves as a measurable indicator of the quantity of mutations within cancer cells and is utilized in the evaluation of genomic instability [28, 29]. MSI, distinguished by heightened mutation rates at genomic microsatellites, is linked to deficiencies in the mismatch repair system. Various types of human cancers have been linked to MSI as a result of deficiencies in mismatch repair. Level 3 RNAseq data and the corresponding clinical information for LUAD were acquired from TCGA. Spearman’s correlation analysis was utilized to assess the association between non-normally distributed quantitative variables. Statistical significance was determined at a p-value threshold of less than 0.05 [14].

The mRNAsi was calculated using the one-class logistic regression (OCLR) algorithm, which incorporated mRNA expression features from gene expression profiles of 11,774 genes [30]. The resulting mRNAsi values were normalized to the [0,1] range through a linear transformation process involving subtraction of the minimum value and division by the maximum value, implemented using the Spearman correlation method [31].

### Expression of RPL22L1 in single cells of LUAD

The Tumor Immune Single-cell Hub 2 (TISCH2) database, accessible at http://tisch.comp-genomics.org/, serves as an scRNA-seq repository tailored for the investigation of the TME [14, 32]. TISCH2 offers comprehensive cell type annotations at the individual cell level, facilitating the examination of the TME in various cancer contexts [33].

### Genomic variants analysis of RPL22L1 in LUAD

RNA sequencing data (level 3), mutation MAF data, and associated clinical information for LUAD were acquired from TCGA. Somatic mutations in LUAD patients were retrieved and visualized utilizing the maftools package within the R software environment. Horizontal histograms depicted a notable prevalence of mutations among individuals diagnosed with LUAD.

### Validation of RPL22L1 expression in the GEO database

In order to confirm the expression of RPL22L1 in LUAD, the dataset GSE87340 was employed, which consisted of 28 tumor samples and 26 normal samples [34].

### Cell culture

Human LUAD cell lines HCC-827, H1299, A549, and H1975 were obtained from Beijing ChosenMed Clinical Laboratory Co., Ltd. These cell lines were cultured in DMEM medium (Sigma, USA) with 10% fetal bovine serum (FBS) and maintained at 37° C with 5% CO_2_ [35].

### Cell transfection

The primer information was as follows:

RPL22L1-siRNA1-SS sequence: GGGAGAAGGUUAAAGUCAAUG,

RPL22L1-siRNA1-AS sequence: UUGACUUUAACCUUCUCCCGU;

RPL22L1-siRNA2-SS sequence: CACAGUUGUUUCUGAGAAACA,

RPL22L1-siRNA2-AS sequence: UUUCUCAGAAACAACUGUGAU;

RPL22L1-siRNA3-SS sequence: CCAGAUUAGUCAAGAUGAAGA;

RPL22L1-siRNA3-AS sequence: UUCAUCUUGACUAAUCUGGAA.

The cancer cells were transfected with Lipofectamine 2000 (Thermo Fisher Scientific, USA) following manufacturer instructions, and the experiment was carried out after 24 h of incubation [36].

### Western blot

The Western blot experiments followed a published protocol, repeated three times. Cells were lysed with RIPA buffer to extract total protein, protein concentration was measured with a BCA kit, and 30 μg of protein were separated using SDS-PAGE and transferred to a PVDF membrane [36].

Subsequently, the membrane was blocked with 5% skimmed milk powder (P0216, Beyotime, China) at room temperature for 1h before the introduction of antibodies for incubation. Finally, color development was achieved using the enhanced chemiluminescence (ECL) chemical hypersensitivity chromogenic reagent kit. Primary antibodies used in this study were rabbit anti-human RPL22L1, mouse MDM2, p-MDM2, p53, and β-actin. The secondary antibody was goat anti-rabbit IgG. Protein band quantification was done with ImageJ software.

### CCK8 and colony formation assay

The CCK-8 assay kit was used to measure cell viability. Cells were seeded in 96-well plates at 2 × 10^3^ cells/well and treated with 10 μL of CCK-8 solution after 0, 24, 48, and 72 h of culture. Absorbance at 450 nm was then measured after a 2 h incubation at 37° C [36].

Cells were digested with 0.25% trypsin and seeded into DMEM medium with 10% FBS at a density of 8 × 10^2^ cells per well. After 2 weeks of culture, colonies were fixed with paraformaldehyde, stained with crystal violet, and counted [36]. Colony formation efficiency was calculated as the ratio of colonies formed to cells seeded, multiplied by 100%.

### Wound healing assay and transwell invasion assay

A549 cells transfected with siRNA were seeded in 6-well plates and observed under a microscope at 0, 24, and 48 h to measure scratch healing area using Image J software.

A549 cells were seeded in a transwell chamber at a density of 3 × 10^4^ cells/well and incubated for 24 h. The inner chamber had 100 μL of cell suspension, while the outer chamber had 600 μL of 1640 medium with 20% FBS. Noninvasive cells in the upper chamber were removed, and the remaining cells on the membrane were fixed with 4% paraformaldehyde and stained with 0.1% crystal violet. Following this, the cells were subjected to examination under a 100 × microscope, and images were captured from five randomly selected fields of view [37].

### Flow cytometry

Quantification of apoptosis levels was performed following the established methodology [18]. The cells were cultured with Annexin-V labeled with FITC (20 μg/mL) and 5 μL PI (50 μg/mL), and subsequently analyzed using a FACSCalibur cytometer [36].

### Tumor xenograft growth assay *in vivo*

Animal experiments followed Nanjing Drum Tower Hospital’s ethics committee guidelines. Caki-1 cells transfected with pcDNA3.1-TPD52 or pcDNA3.1 were injected into female Balb/C nude mice [38]. Tumor volume was measured every 5 days using a specific formula. After approximately 20 days, tumors were weighed after mice were anesthetized [38].

### Immunohistochemistry

The study used FFPE samples and incubated tumor sections with rabbit polyclonal antibodies against RPL22L1 at a dilution of 1/100 overnight at 4° C [39]. The sections were then conjugated with HRP-Sheep Anti-Rabbit IgG-HRP-Sheep Anti-Mouse IgG antibody at a dilution of 1:500 and treated with DAB [39]. The Elivision plus kit for IHC was used [39].

### Statistical analysis

The analytical methods and R packages utilized in this study were executed using R software version 4.0.3 (R Foundation for Statistical Computing, 2020) [40]. Statistical analyses involved the application of the Wilcoxon signed rank test and the t-test. Results with p-values below 0.05 were considered statistically significant [34, 41, 42].

### Data availability

All data generated or analyzed during this study are included in this article.

## RESULTS

### Aberrant expression of RPL22L1 in LUAD and correlation with clinical characteristics

**Figure 1:**
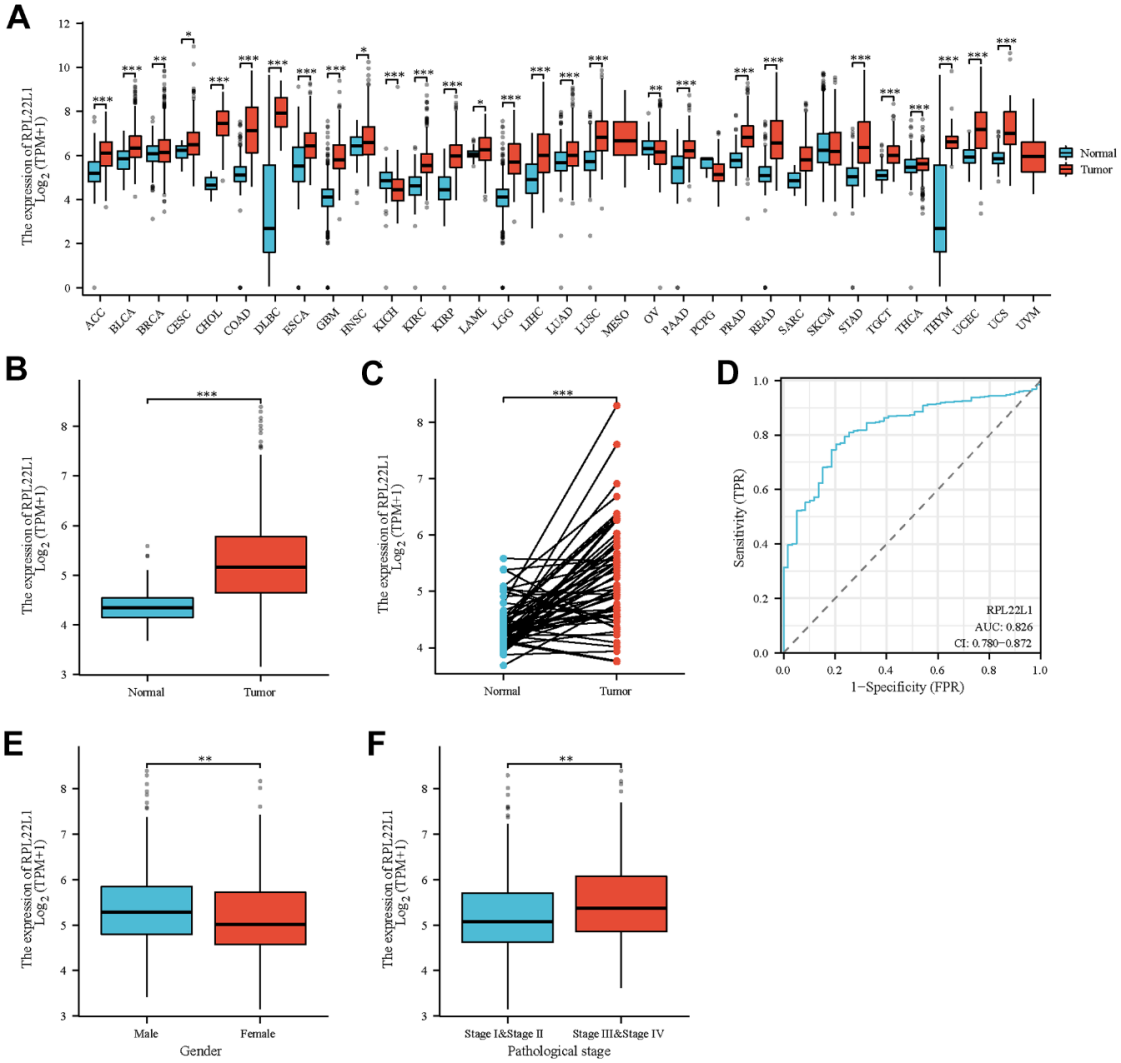
**Abnormal expression of RPL22L1 in LUAD and its correlation with various clinical features.** (**A**) Differential expression of RPL22L1 in pan cancer and normal tissues. (**B**) Specific differential expression of RPL22L1 in LUAD and normal lung tissue. (**C**) Differential expression of RPL22L1 in LUAD and its paired normal lung tissues. (**D**) The efficacy of RPL22L1 expression in distinguishing LUAD tissue from non-tumor tissue is shown by the ROC curve. (**E**, **F**) represent T stage and pathological stage, respectively. The statistical significance is represented by *, p < 0.05, * *, p < 0.01, * * *, and p < 0.001, respectively.

The clinical characteristics of TCGA-LUAD, such as T stage, N stage, pathologic stage, gender, age, anatomic neoplasm subdivision, and RPL22L1, were gathered for analysis in this study, as presented in Supplementary Table 1. Figure 1A and Supplementary Table 2 illustrates a comprehensive examination of RPL22L1 expression across various cancer types, indicating a notable up-regulation in 26 tumors and a significant down-regulation in 2 tumors. Thus, it can be deduced that the aberrant expression of RPL22L1 is intricately linked to tumor progression. Data depicted in Figure 1B indicate a statistically significant increase in the expression level of RPL22L1 in LUAD tumor tissues compared to unpaired normal tissues (p < 0.001). The results presented in Figure 1C reveal a notable elevation in the expression level of RPL22L1 in LUAD tumor tissues in comparison to corresponding normal tissues (p < 0.001). Figure 1D illustrates an area under the curve (AUC) value of 0.826 for RPL22L1, suggesting its potential as a dependable biomarker for distinguishing LUAD from normal liver tissues.

In order to examine the clinical attributes linked to RPL22L1, an analysis was conducted to evaluate the relationship between the expression of RPL22L1 and diverse factors in individuals diagnosed with LUAD. The results, as delineated in Supplementary Table 3 and Figure 1E, 1F, demonstrate a notable correlation between elevated levels of RPL22L1 and both pathological stage (p = 0.008) and gender (p = 0.004) among LUAD patients.

### RPL22L1 was significantly associated with prognosis in TCGA-LUAD patients

**Figure 2:**
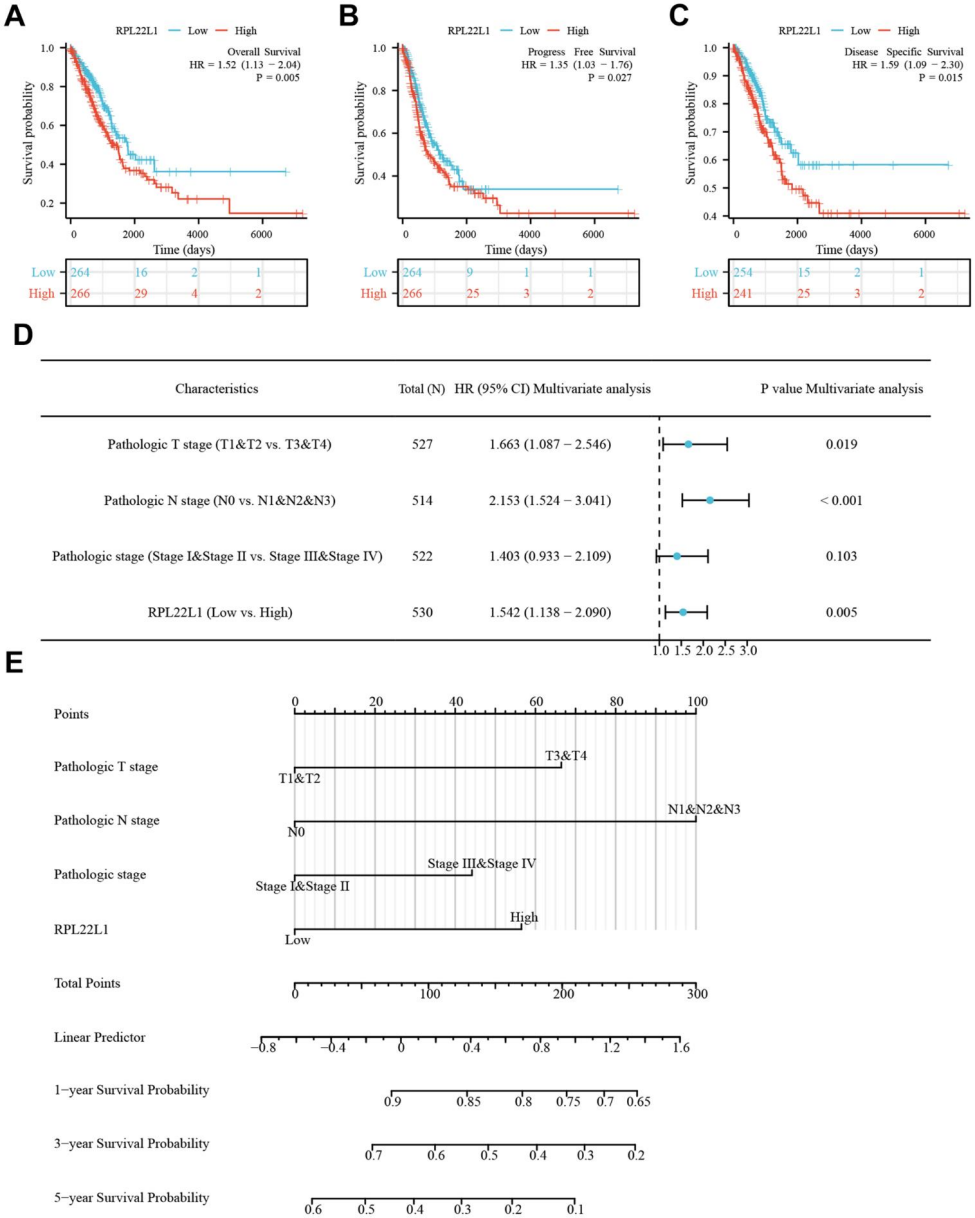
**RPL22L1 was an independent variable for predicting OS in LUAD.** (**A**) OS. (**B**) PFS. (**C**) DSS. (**D**) Forest plot display of the results of the multivariate Cox regression analysis of RPL22L1 and clinical characteristics in LUAD. (**E**) Nomograms were developed to estimate the likelihood of OS at 1-, 3-, and 5-year intervals in patients with LUAD.

Moreover, an examination was conducted on the OS, PFS, and DSS of patients diagnosed with LUAD. It was observed that patients exhibiting elevated levels of RPL22L1 experienced significantly poorer OS (p = 0.005, Figure 2A), PFS (p = 0.027, Figure 2B), and DSS (p = 0.015, Figure 2C). These findings suggest that RPL22L1 may serve as a potential prognostic marker for predicting unfavorable outcomes in LUAD, thereby offering valuable insights for the advancement of treatment strategies for this disease. Univariate and multifactorial Cox regression analyses revealed significant associations between pathologic T stage, N stage, pathological stage, and RPL22L1 expression with OS in patients with LUAD (Figure 2D and Supplementary Table 4). Specifically, the results indicated that tumor status and RPL22L1 expression are independent risk factors influencing the prognosis of LUAD patients. Integrating RPL22L1 expression with clinical variables facilitated the creation of column line plots, depicted in Figure 2E, which enabled the prediction of 1-, 3- and 5-year survival probabilities among LUAD patients.

### Pathways involved in RPL22L1 in TCGA-LUAD

**Figure 3:**
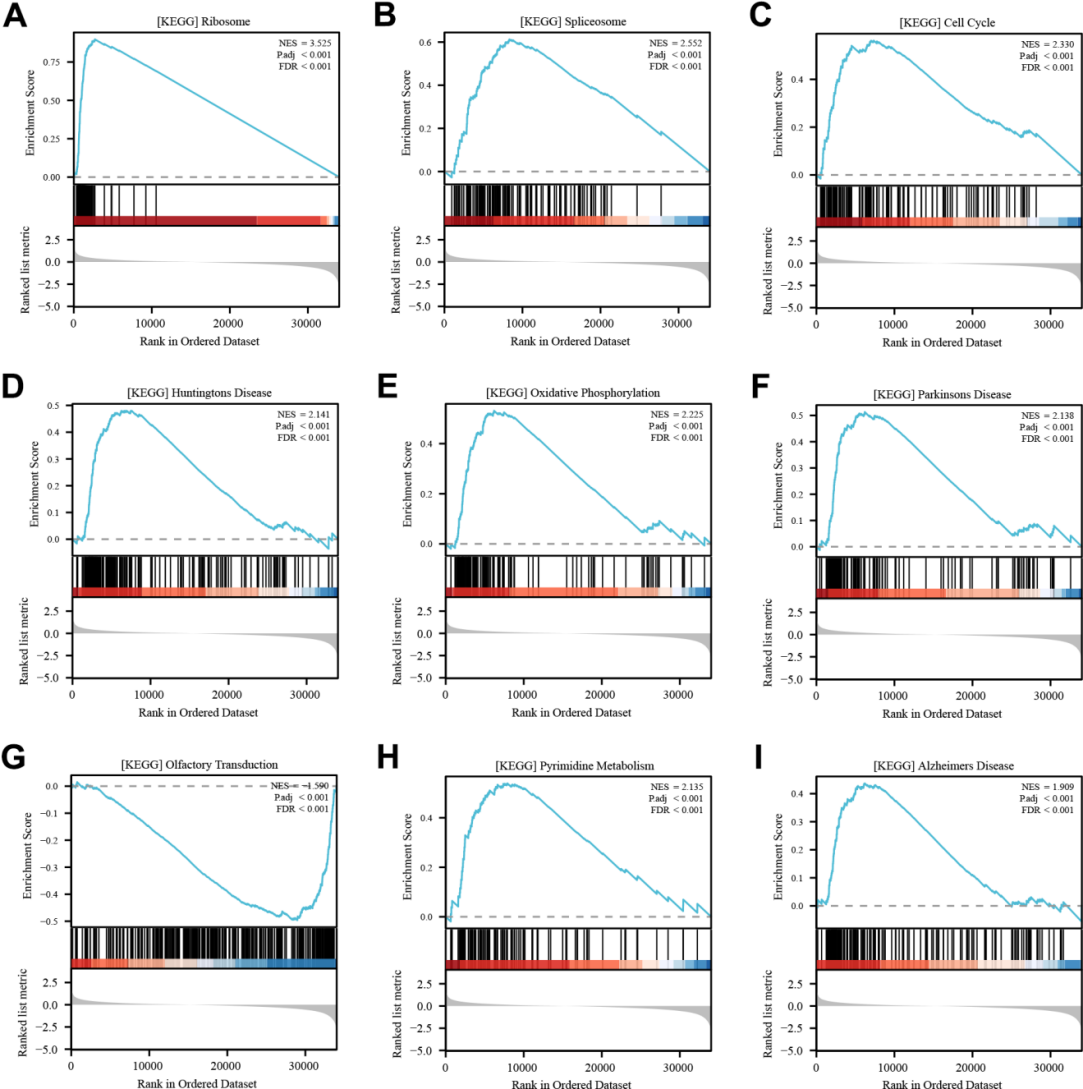
**Enrichment analysis of RPL22L1 (GSEA).** (**A**) Ribosome. (**B**) Spliceosome. (**C**) Cell cycle. (**D**) Huntington’s disease. (**E**) Oxidative phosphorylation. (**F**) Parkinsons disease. (**G**) Olfactory transduction. (**H**) Pyrimidine metabolism. (**I**) Alzheimer's disease. NES, which stands for normalized ES, and FDR, which stands for false discovery rate, were utilized in this study.

To elucidate the potential mechanism underlying the role of RPL22L1 in LUAD, a set of genes related to RPL22L1 was obtained by GSEA analysis. The pathways significantly associated with RPL22L1 included ribosome, spliceosome, cell cycle, oxidative phosphorylation, Parkinson’s disease, olfactory transduction, and pyrimidine metabolism (Figure 3).

### RPL22L1 was significantly associated with immune infiltration, immune score, and immune checkpoint genes

**Figure 4:**
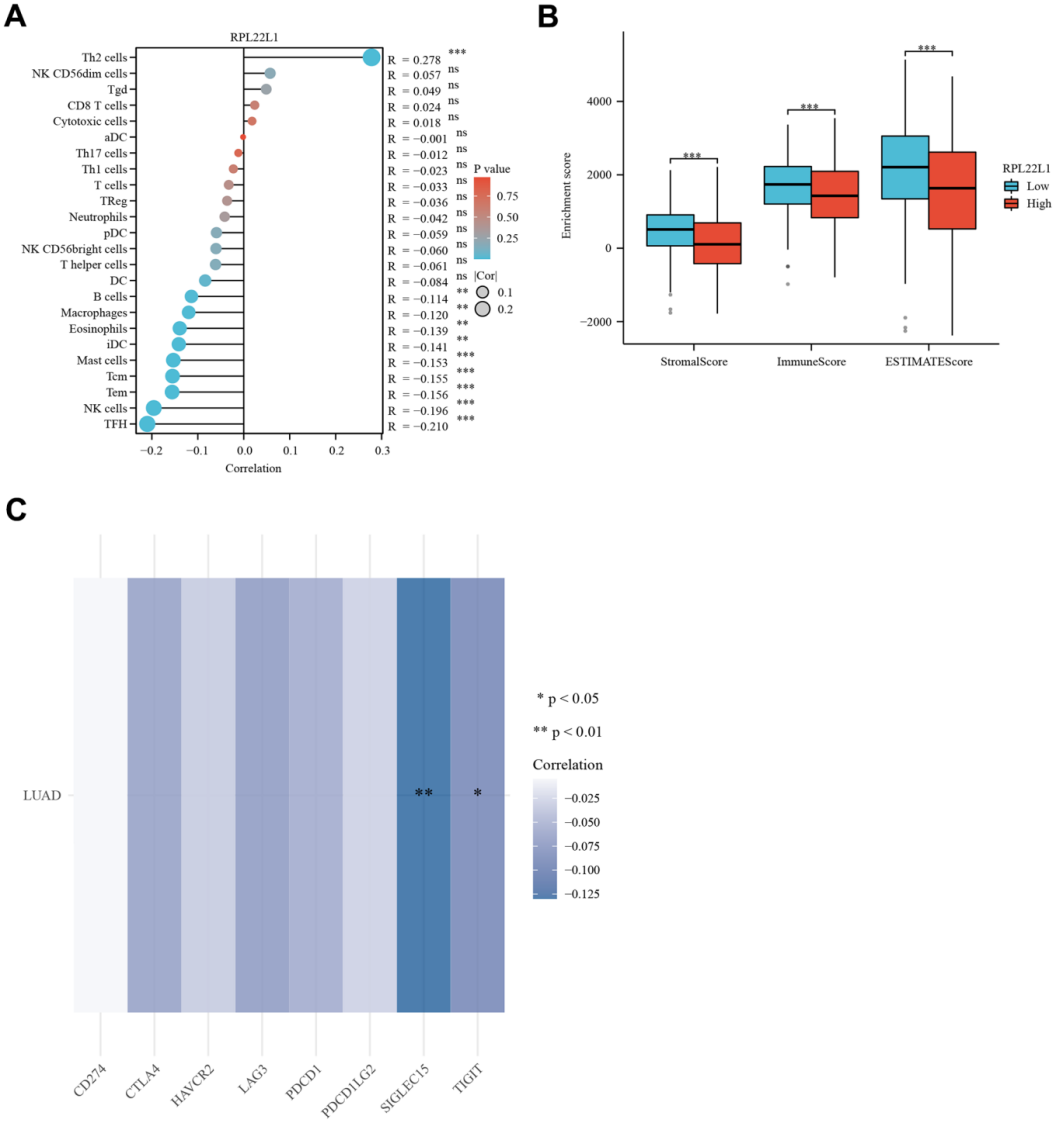
**RPL22L1 expression was associated with immune infiltration, immune score, and immune checkpoint genes in LUAD.** (**A**) Lollipop plot. (**B**) Grouped comparison plots. (**C**) Check point genes. Statistical significance was denoted by asterisks, with *, **, and *** representing p-values less than 0.05, 0.01, and 0.001, respectively.

Figure 4A demonstrated a notable positive correlation (p < 0.0001) between the expression of RPL22L1 and Th2 cells. The data presented indicates a significant inverse relationship between the expression of RPL22L1 and various immune cell types, including B cells, Eosinophils, iDC, Macrophages, Mast cells, NK cells, Tcm, Tem, and TFH (p < 0.05 for all). The group with low RPL22L1 expression exhibited elevated stromal score, immune score, and ESTIMATE score (Figure 4B). Figure 4C demonstrates a notable negative correlation between RPL22L1 expression and SIGLEC15 and TIGIT.

### Expression of RPL22L1 was significantly correlated with TMB/MSI and CSCs

**Figure 5:**
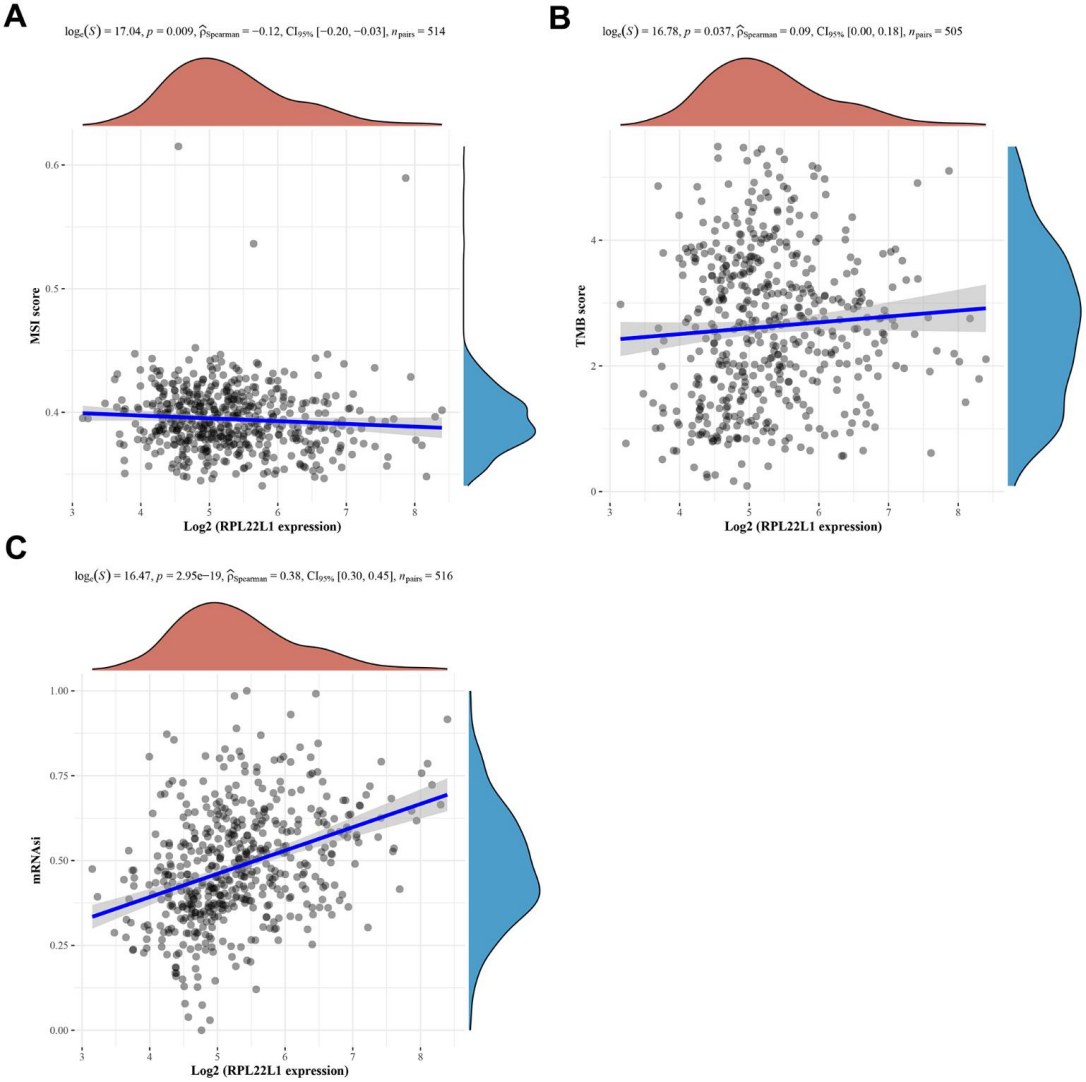
**Spearman correlation analysis of MSI/TMB and mRNAsi and RPL22L1 gene expression in LUAD.** (**A**) MSI. (**B**) TMB. (**C**) mRNAsi. The figure displays the gene expression distribution on the horizontal coordinates and the mRNAsi distribution on the vertical coordinates. The density curve on the right side represents the trend of the mRNAsi distribution, while the density curve on the upper side represents the trend of the gene expression distribution. The uppermost values in the figure indicate the correlation p-value, correlation coefficient, and the method used for correlation calculation.

In LUAD, the expression of RPL22L1 exhibited a notable inverse association with MSI and a marked positive relationship with TMB, as depicted in Figure 5A, 5B, respectively.

The progression of cancer entails the gradual deterioration of a distinct phenotype and the adoption of traits reminiscent of progenitor/stem cells. To assess the degree of stemness in a given sample, we utilized OCLR machine learning to compute a stemness index derived from the sample’s transcriptome. Our analysis, depicted in Figure 5C, demonstrated a statistically significant correlation between the expression of RPL22L1 and cancer stem cells (p = 2.95e-19).

### RPL22L1 exhibited substantial upregulation in LUAD single cells and demonstrated a correlation with immune infiltration

**Figure 6:**
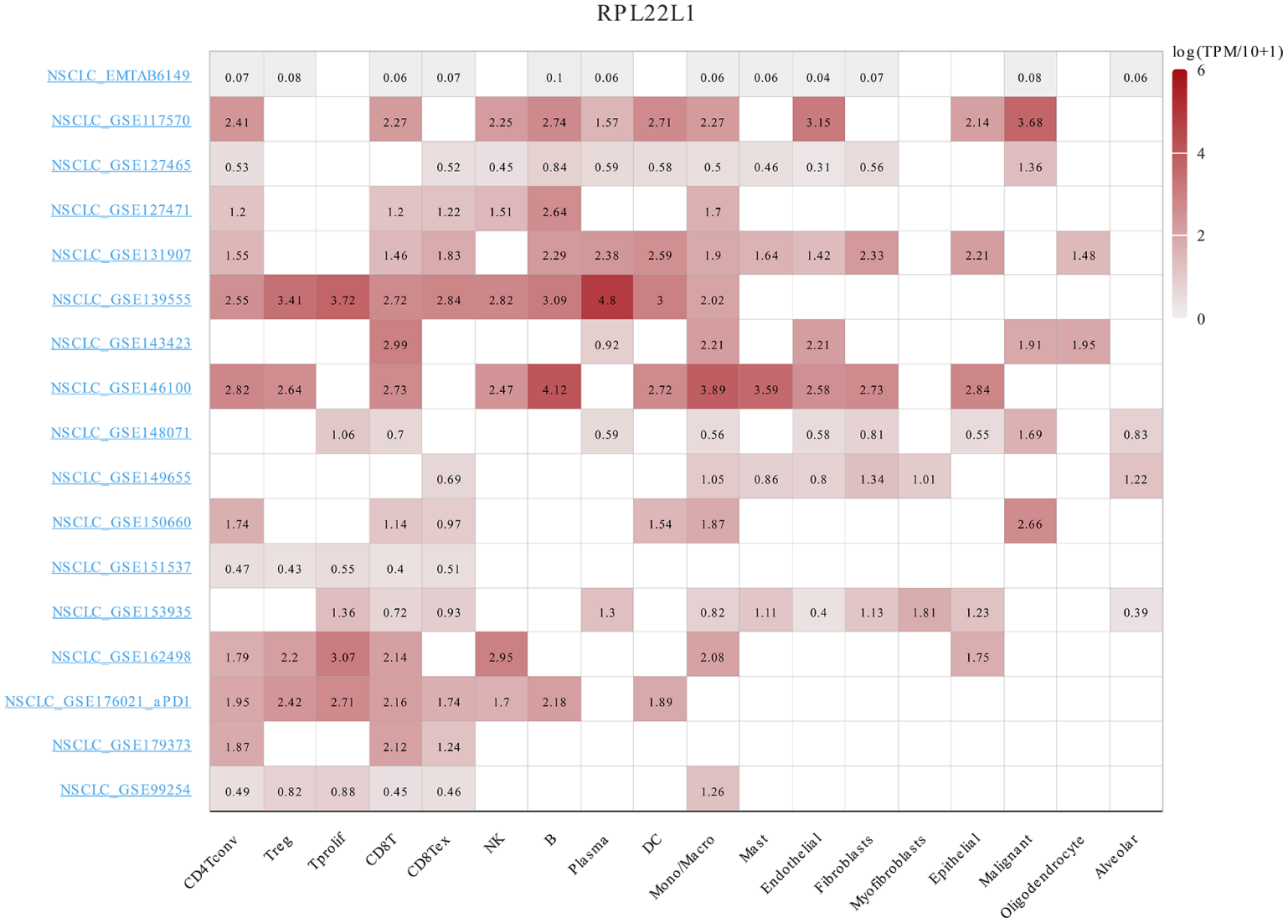
The expression of RPL22L1 was related to immune infiltration in LUAD single cells.

As shown in Figure 6, the RPL22L1 gene was upregulated in multiple individual cells of LUAD, including CD4Tconv, Treg, Tprolif, CD8T, CD8Tex, NK, B, Plasma, DC, Mono/Macro, Mast, Endothelial, Fibroblasts, Myofibroblasts, Epithelial, Malignant, Oligodendrocyte, and Alveolar.

### Somatic variants of RPL22L1 in LUAD

**Figure 7:**
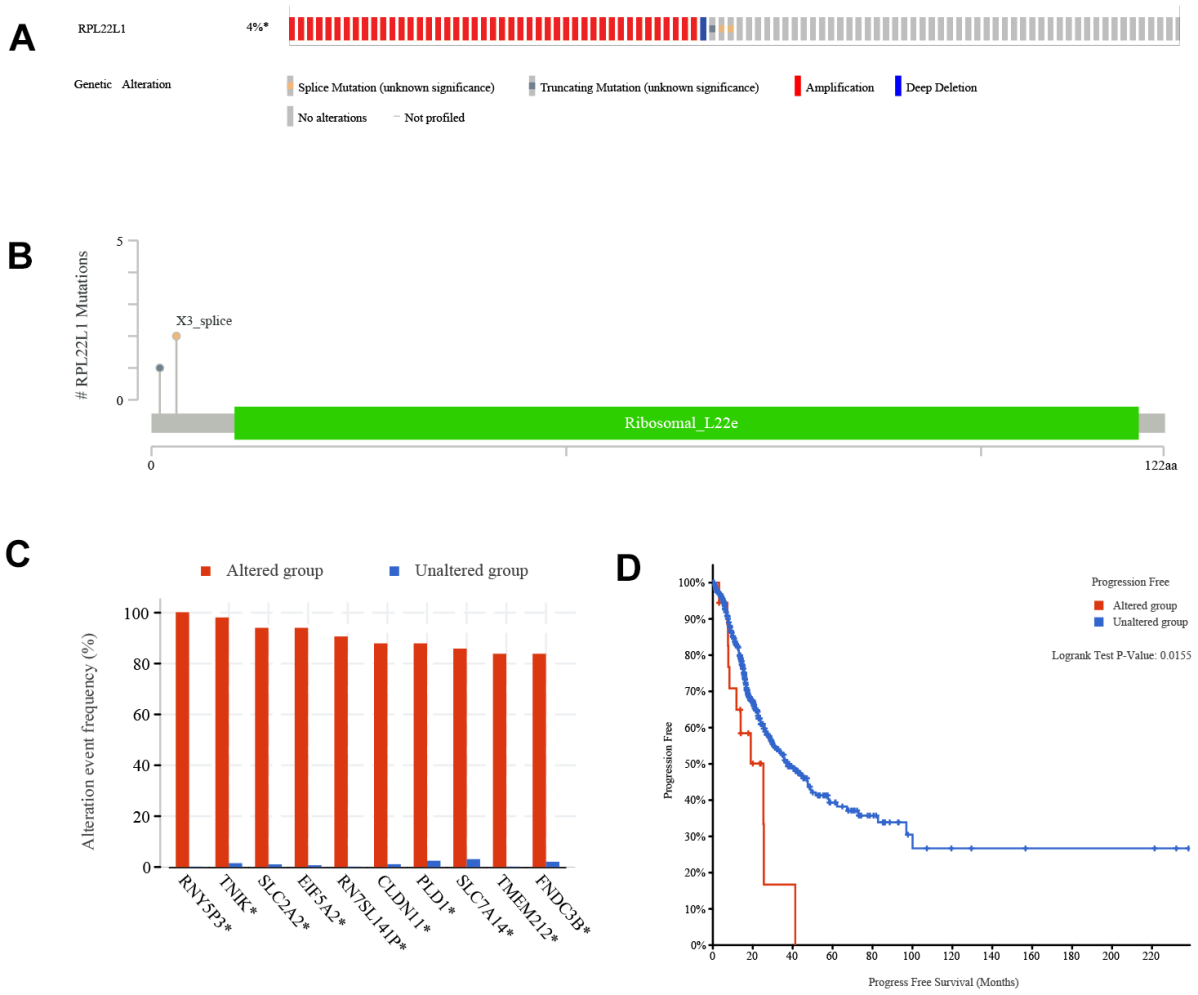
**Genetic alterations of RPL22L1 in LUAD.** (**A**) This study examines the presence of structure variants, mutations, and copy number alterations in the gene RPL22L1. (**B**) The aim of this research is to provide a comprehensive description of the various types, number, and location of mutations occurring in the gene RPL22L1. (**C**) The frequency of gene alterations is assessed in both the RPL22L1 altered and unaltered groups, in order to determine their association with the gene RPL22L1. (**D**) A comparative analysis of progression free survival is conducted between the RPL22L1 altered group and the RPL22L1 unaltered group in pan cancer cases.

According to the data presented in Figure 7A, the frequency of variation in RPL22L1 was found to be 4%, with amplification, splice mutation, deep deletion, and truncation mutation being the types of variation observed. Figure 7B illustrates that the variant sites of RPL22L1 included 2 splices and 1 truncating mutation. Figure 7C demonstrates that gene alterations in RNY5P3, TNIK, SLC2A2, EIF5A2, RN7SL141P, CLDN11, PLD1, SLC7A14, TMEM212 and FNDC3B were more prevalent in the altered group of RPL22L1 compared to the unaltered group of RPL22L1. Figure 7D reveals a significant decrease in PFS among LUAD patients in the altered group RPL22L1 (n = 18) compared to the unaltered group RPL22L1 (n = 487), with a p-value of 0.0155.

### RPL22L1 was significantly overexpressed in LUAD

**Figure 8:**
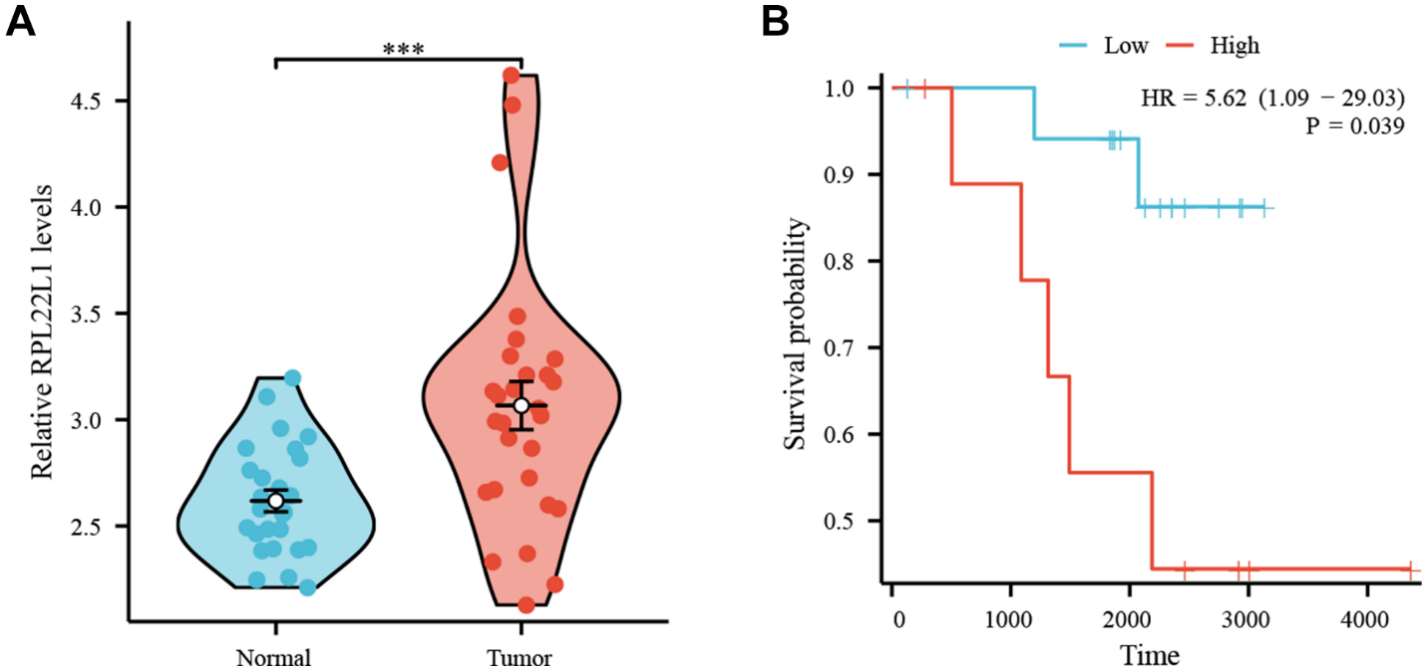
**High expression of RPL22L1 in GSE87340 suggests a poor prognosis.** (**A**) The expression of RPL22L1 in GSE87340. (**B**) OS.

Figure 8A demonstrates a significant upregulation of RPL22L1 expression in LUAD tissues compared to normal lung tissues (p < 0.001). Figure 8B illustrates a correlation between high RPL22L1 expression and poor OS (p = 0.039).

### RPL22L1 promotes cell proliferation, migration, and invasion in LUAD cell lines

**Figure 9:**
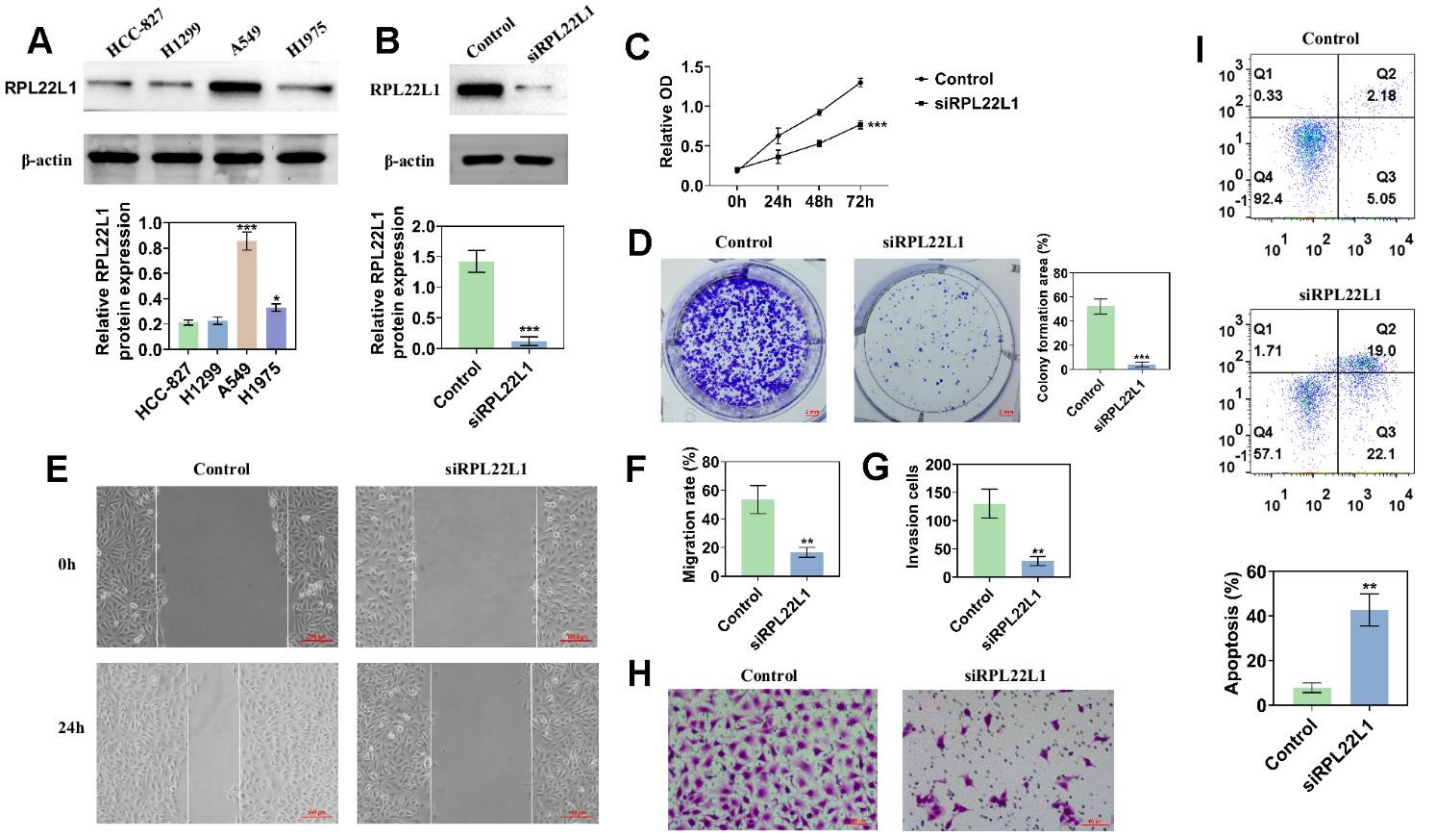
**The knockdown of RPL22L1 in A549 cells was found to enhance their proliferation, migration, and invasion.** (**A**) Western blot analysis was conducted to assess the expression of RPL22L1 in LUAD cell lines. (**B**) The knockdown efficiency of RPL22L1 in A549 cells was evaluated using Western blot analysis. (**C**) The impact of RPL22L1 knockdown on the viability of A549 cells was determined through a cell viability assay. (**D**) Cloning experiments were performed to investigate the effect of RPL22L1 knockdown on the proliferation ability of A549 cells. (**E**, **F**) The wound healing assay demonstrated the impact of PRL22L1 knockdown on the migratory behavior of A549 cells. (**G**, **H**) Transwell assays revealed that RPL22L1 knockdown had the potential to decrease the invasive capabilities of A549 cells. (**I**) Flow cytometry analysis provided insights into the influence of PRL22L1 knockdown on apoptosis in A549 cells. Statistical significance was denoted by *p < 0.05, **p < 0.01, and ***p < 0.001.

Figure 9A shows the expression of RPL22L1 in lung adenocarcinoma cell lines. The highest expression level of RPL22L1 was observed in the A549 cell line. Following silencing of RPL22L1, there was a significant decrease in the expression of RPL22L1 (Figure 9B). The group in which RPL22L1 was silenced exhibited a reduction in cell activity compared to the control group (Figure 9C). Compared to the control group, the RPL22L1 silencing group showed weaker proliferation, migration, and invasion abilities (Figure 9D–9H). Furthermore, the RPL22L1 silencing group demonstrated an increase in apoptosis compared to the control group (Figure 9I).

### RPL22L1 increases the growth of xenograft tumors *in vivo*

**Figure 10:**
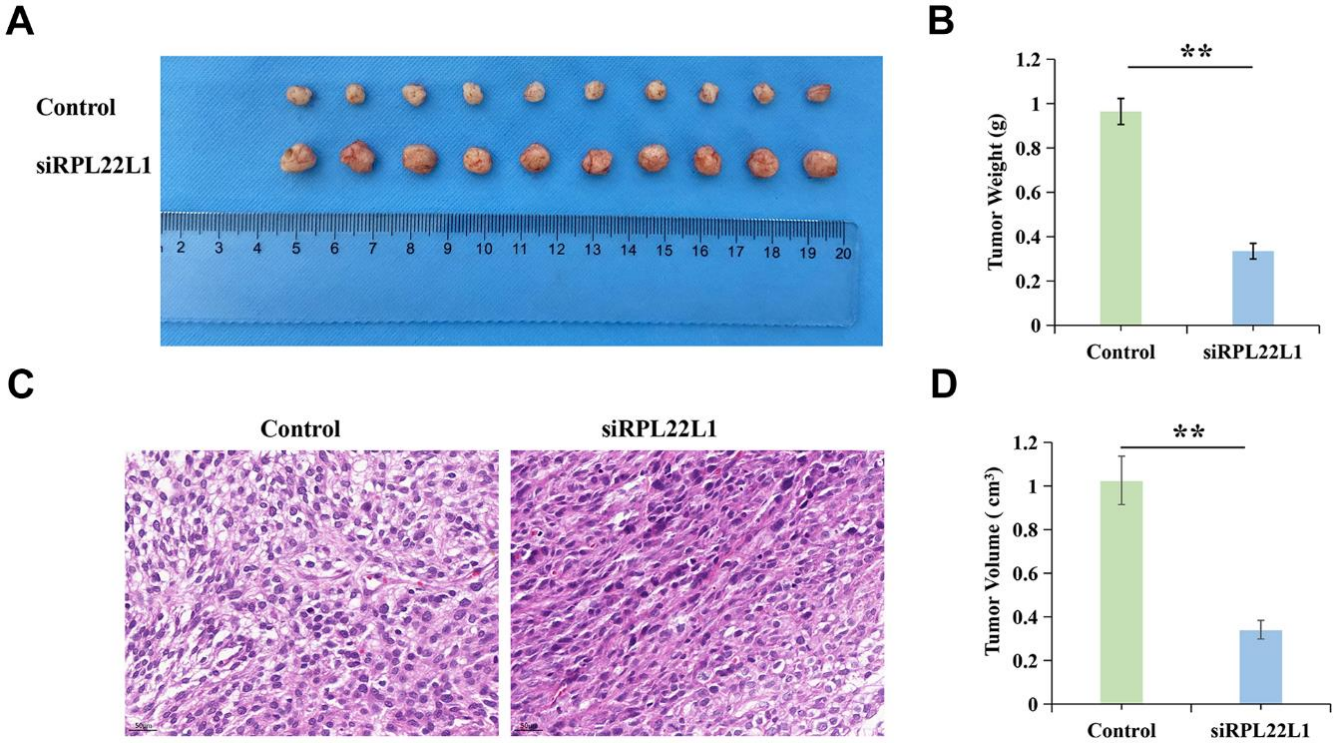
**RPL22L1 can increase the growth of xenograft tumors *in vivo*.** (**A**) Images of tumors in the control group and siRPL22L1 group. (**B**) Compared with the control group, the siRPL22L1 group showed a significant decrease in tumor weight. (**C**) Immunohistochemical images of the control group and siRPL22L1 group. (**D**) The siRPL22L1 group exhibited a statistically significant reduction in tumor volume when compared to the control group. **, P<0.01.

In order to investigate the impact of RPL22L1 on LUAD growth *in vivo*, xenograft tumors were utilized in Balb/C nude mice. Notably, the suppression of RPL22L1 through silencing resulted in a significant inhibition of tumor weight and volume compared to the vector group (Figure 10A, 10B, 10D). Furthermore, we observed fewer invasions of neighboring tissues by A549 cells transfected with siRPL22L1 (Figure 10C).

### RPL22L1 can activate MDM2/p53

**Figure 11:**
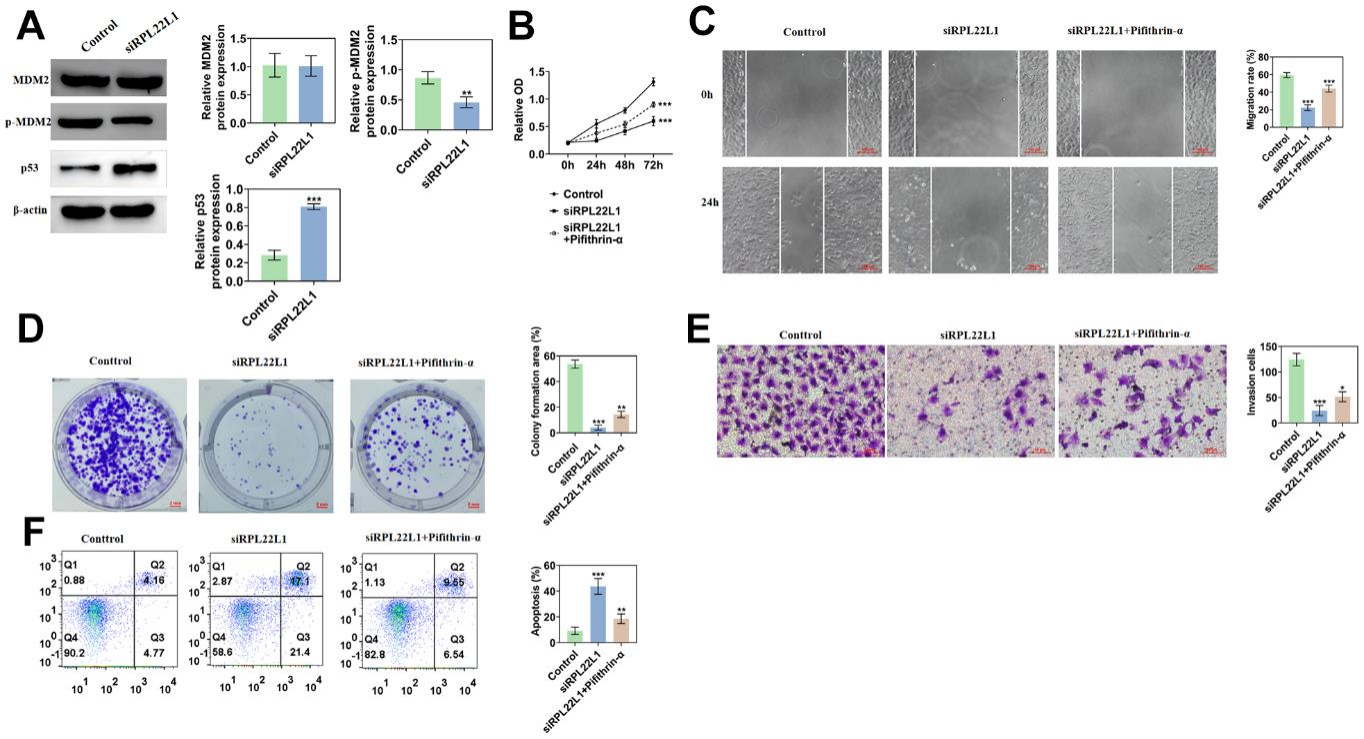
**RPL22L1 can activate MDM2/p53.** (**A**) Western blotting showed the effect of PRL22L1 knockdown on the expression of MDM2/p53. (**B**) The CCK-8 experiment demonstrated the impact of RPL22L1 knockdown in combination with pifithrin-α on the proliferative capacity of A549 cells. (**C**) The wound healing assay revealed the influence of PRL22L1 knockdown in conjunction with pifithrin-α on the migratory ability of A549 cells. (**D**) The cloning experiments exhibited the effect of PRL22L1 knockdown in combination with pifithrin-α on the proliferation capability of A549 cells. (**E**) The transwell experiment demonstrated the impact of PRL22L1 knockdown in conjunction with pifithrin-α on the invasive potential of A549 cells. (**F**) The flow cytometry analysis revealed the influence of PRL22L1 knockdown in combination with pifithrin-α on the apoptotic ability of A549 cells. *, p < 0.05, **, p < 0.01; ***, p < 0.001.

In contrast to the control group, the RPL22L1 silenced group exhibited no alteration in MDM2 expression, but a marked decrease in p-MDM2 expression and a significant increase in p53 expression (Figure 11A). The group with low expression, when compared to the control group, displayed diminished cellular activity, reduced proliferation, migration, and invasion capacities, and enhanced apoptosis (Figure 11B–11F). In comparison to the low expression group, the group exhibiting low expression combined with an inhibitor demonstrated heightened cellular activity, enhanced proliferation, migration, and invasion capabilities, as well as decreased apoptosis (Figure 11B–11F).

## DISCUSSION

The precise function of RPL22L1 in LUAD remains uncertain. Recent research has revealed a significant upregulation of RPL22L1 expression at the mRNA and protein levels in both LUAD tissues and cell lines. Knockdown of RPL22L1 has been shown to suppress the activity, proliferation, migration, and invasion of LUAD cells, induce apoptosis, and attenuate tumor growth in LUAD xenografts in mice. Mechanistically, the inhibition of RPL22L1 has been found to markedly reduce MDM2 phosphorylation and increase P53 levels in LUAD cells.

Lung cancer is a prevalent global disease, with LUAD accounting for approximately 40% of the reported cases [2]. The present study introduces a robust biomarker, namely RPL22L1, which exhibits elevated expression in LUAD patients, aligning with its increased expression in PRAD, HCC, CRC, KIRC, and OC [7, 9–13]. Our study demonstrates a significant increase in the expression of RPL22L1 at the mRNA and protein levels in both LUAD tissues and cell lines. Inhibition of RPL22L1 significantly impeded the activity, proliferation, migration, and invasion of LUAD cells, while also promoting apoptosis and reducing the growth of LUAD xenografts. These findings strongly support the notion that RPL22L1 acts as a crucial factor in promoting tumor progression in LUAD. In particular, this study represents the initial identification of a notable upregulation of RPL22L1, the elucidation of its biological function and the assessment of its impact on the immune status of LUAD patients.

This study has contributed further insights into the relationship between RPL22L1 and prognosis, revealing that elevated RPL22L1 expression is associated with a less favorable prognosis in LUAD. Through univariate and multivariate Cox regression analyses, the expression of RPL22L1 was determined to be an independent prognostic factor in LUAD. However, it is important to acknowledge that a single factor cannot provide a comprehensive and precise assessment of the prognosis of the tumor. Therefore, to address this limitation, a column line graph was employed that incorporates RPL22L1 expression and pertinent clinical data. Therefore, there is compelling evidence that RPL22L1 is an independent predictor of the prognosis of LUAD.

The research introduced the immune-based assay “Immunoscore” to measure T-cell infiltration *in situ*, demonstrating its superior efficacy over TNM classification in individuals with cancer [43]. Our results reveal a notable reduction in stromal score, immune score, and ESTIMATE score among those in the high-expression group of RPL22L1. Patients with elevated RPL22L1 expression in LUAD exhibit diminished immune and stromal elements in their tumor microenvironment, suggesting a potential association between heightened RPL22L1 expression and compromised immune status in LUAD patients.

The investigation of immune infiltration in LUAD has emerged as a significant research focus [44]. Immunotherapy has demonstrated potent antitumor effects across various cancer types [45]. While immune checkpoint inhibitors (ICIs) have shown promising efficacy in LUAD, ongoing research is exploring additional immunotherapeutic approaches for patients with this disease [46]. The primary objective of this study was to examine the correlation between RPL22L1 expression and immune responses in patients with LUAD. Our findings suggest that RPL22L1 expression in LUAD is associated with the infiltration of diverse immune cell populations.

The documented involvement of immune checkpoints in physiological immune responses, their up-regulation in the TME of various malignant tumors, and subsequent induction of antitumor immune responses have led to significant improvements in patient prognosis in clinical settings through the use of ICIs [47]. Our findings demonstrate a negative correlation between RPL22L1 and the SIGLEC15 and TIGIT immune checkpoints indicating the significant immunomodulatory role of RPL22L1 and its potential as a biomarker for immunotherapy.

TMB is an emerging immunotherapy biomarker in addition to PD-L1 expression. MSI, as the most important biomarker for the precise diagnosis and treatment of pan tumors, is also closely related to the incidence of LUAD. Although the research on MSI in lung cancer is not yet mature compared to CRC, and the incidence of MSI-H varies between different studies (0.4% -29%), MSI detection is increasingly valued in patients with LUAD. The study investigated the association between RPL22L1 expression and MSI and TMB in LUAD. The results indicated a significant relationship between RPL22L1 expression and MSI and TMB in LUAD. Specifically, RPL22L1 expression in LUAD was positively correlated with TMB and negatively correlated with MSI, suggesting that RPL22L1 may serve as a potential biomarker for assessing the immunogenicity of LUAD.

RPL22L1 promotes resistance to temozolomide in glioblastoma by activating STT3 [8]. RPL22L1 plays a significant role in facilitating the proliferation and invasion of PRAD cells via the PI3K/Akt/mTOR pathway [10]. RPL22L1 is implicated in activating ERK to promote atypical epithelial mesenchymal transition (EMT) progression [7]. RPL22L1 is essential for maintaining the aggressive OC phenotype and for triggering cell metastasis by inducing EMT [13]. The regulatory mechanism governing RPL22L1 expression in LUAD remains unexplored. As a ‘genome guardian’, P53 plays a crucial role in the cellular stress response network, including monitoring ribosomal biogenesis [48]. MDM2, an E3 ubiquitin ligase, plays a crucial role in targeted protein-dependent proteome degradation [49]. It mediates the rapid degradation of p53 and regulates the stability and function of the P53 protein by directly inhibiting its transcriptional activity through binding to the transcriptional activation regions and target P53 [50, 51]. The MDM2-P53 pathway is significantly involved in tumor progression [52]. LINC01426 exacerbates the malignant progression of glioma by modulating the miR-661/MDM2/P53 axis [53]. Current research shows that silencing RPL22L1 significantly down-regulates the expression level of p-MDM2 in A549 cells and up-regulates the protein expression level of P53. Notably, RPL22L1 is implicated in enhancing the growth and metastasis of LUAD by inhibiting the MDM2/P53 signaling pathway.

Consequently, these findings propose that targeting RPL22L1 could offer potential as a therapeutic strategy for managing LUAD.

## CONCLUSIONS

In LUAD, RPL22L1 exhibited significant up-regulation and displayed a notable correlation with unfavorable OS, PFS, and DSS. RPL22L1 was associated with the TME, TMB/MSI, and mRNAsi. Patients with LUAD in the altered RPL22L1 group had a shorter PFS compared to those in the unaltered RPL22L1 group. Mechanistically, RPL22L1 was found to enhance cell proliferation, migration, and invasion of LUAD tumors through activation of the MDM2/p53 signaling pathway. These results suggest that RPL22L1 may serve as a promising prognostic marker and therapeutic target for immunotherapy in LUAD.

## Supplementary Material

Supplementary Tables
