# Supplementary material for: RPL22L1 is a novel biomarker for prognosis and immune infiltration in lung adenocarcinoma, promoting the growth and metastasis of LUAD cells by inhibiting the MDM2/P53 signaling pathway
Source: Aging (Albany NY). 2024 Aug 28;16(17):12392–413. doi: 10.18632/aging.206096 (PMC11424578; doi:10.18632/aging.206096)
Supplement: Supplementary Tables [file aging-16-206096-s001.pdf]

SUPPLEMENTARY TABLES

Supplementary Table 1. The correlation between the expression of RPL22L1 in LUAD and clinical features.

Characteristics	Overall	Low expression of RPL22L1	High expression of RPL22L1	p-value	Statistic	Method
n	539	269	270			
Pathologic T stage, n (%)				0.136	2.225	Chisq test
T1&T2	468(87.3%)	238 (44.4%)	230 (42.9%)			
T3&T4	68(12.7%)	28 (5.2%)	40 (7.5%)			
Pathologic N stage, n (%)				0.263	1.253	Chisq test
N0	350(66.9%)	176 (33.7%)	174 (33.3%)			
N1&N2&N3	173(33.1%)	78 (14.9%)	95 (18.2%)			
Pathologic stage, n (%)				0.011	6.491	Chisq test
Stage I&Stage II	421(79.3%)	222 (41.8%)	199 (37.5%)			
Stage III&Stage IV	110(20.7%)	43 (8.1%)	67 (12.6%)			
Gender, n (%)				0.001	10.511	Chisq test
Female	289(53.6%)	163 (30.2%)	126 (23.4%)			
Male	250(46.4%)	106 (19.7%)	144 (26.7%)			
Age, n (%)				0.860	0.031	Chisq test
<= 65	257(49.4%)	127 (24.4%)	130 (25%)			
> 65	263(50.6%)	132 (25.4%)	131 (25.2%)			
Anatomic neoplasm subdivision, n (%)				0.218	1.519	Chisq test
Left	207(39.5%)	110 (21%)	97 (18.5%)			
Right	317(60.5%)	151 (28.8%)	166 (31.7%)			

Supplementary Table 2. Abbreviations for 33 types of tumors in TCGA.

Full name of tumors	Abbreviation of tumors
Adrenocortical carcinoma	ACC
Bladder urothelial carcinoma	BLCA
Breast invasive carcinoma	BRCA
Cervical squamous cell carcinoma and endocervical adenocarcinoma	CESC
Cholangiocarcinoma	CHOL
Colon adenocarcinoma	COAD
Lymphoid neoplasm diffuse large B-cell lymphoma	DLBC
Esophageal carcinoma	ESCA
Glioblastoma multiforme	GBM
Head and neck squamous cell carcinoma	HNSC
Kidney chromophobe	KICH
Kidney renal clear cell carcinoma	KIRC
Kidney renal papillary cell carcinoma	KIRP
Acute myeloid leukemia	LAML
Brain lower grade glioma	LGG
Liver hepatocellular carcinoma	LIHC
Lung adenocarcinoma	LUAD
Lung squamous cell carcinoma	LUSC
Mesothelioma	MESO
Ovarian serous cystadenocarcinoma	OV
Pancreatic adenocarcinoma	PAAD
Pheochromocytoma and paraganglioma	PCPG
Prostate adenocarcinoma	PRAD
Rectum adenocarcinoma	READ
Sarcoma	SARC
Skin cutaneous melanoma	SKCM
Stomach adenocarcinoma	STAD
Testicular germ cell tumors	TGCT
Thyroid carcinoma	THCA
Thymoma	THYM
Uterine corpus endometrial carcinoma	UCEC
Uterine carcinosarcoma	UCS
Uveal melanoma	UVM

Supplementary Table 3. The expression of RPL22L1 in LUAD is related to clinical features (logical analysis).

Characteristics	Total (N)	OR (95% CI)	P-value
Pathologic T stage (T3&T4 vs. T1&T2)	536	1.478 (0.883 - 2.476)	0.137
Pathologic N stage (N1&N2&N3 vs. N0)	523	1.232 (0.855 - 1.776)	0.263
Pathologic stage (Stage III&Stage IV vs. Stage I&Stage II)	531	1.738 (1.133 - 2.667)	0.011
Gender (Male vs. Female)	539	1.757 (1.248 - 2.474)	0.001
Age (> 65 vs. <= 65)	520	0.970 (0.687 - 1.367)	0.86
Anatomic neoplasm subdivision (Right vs. Left)	524	1.247 (0.878 - 1.771)	0.218

Supplementary Table 4. Univariate and multivariate analysis (Cox regression) of clinical features of LUAD and DSS.

Characteristics	Total (N)	Univariate analysis		Multivariate analysis	
		HR (95% CI)	P-value	HR (95% CI)	P-value
Pathologic T stage (T1&T2 vs. T3&T4)	527	2.352 (1.614 - 3.426)	< 0.001	1.663 (1.087 - 2.546)	0.019
Pathologic N stage (N0 vs. N1&N2&N3)	514	2.547 (1.904 - 3.407)	< 0.001	2.153 (1.524 - 3.041)	< 0.001
Pathologic stage (Stage I&Stage II vs. Stage III&Stage IV)	522	2.710 (1.994 - 3.685)	< 0.001	1.403 (0.933 - 2.109)	0.103
Gender (Female vs. Male)	530	1.087 (0.816 - 1.448)	0.569		
Age (<= 65 vs. > 65)	520	1.216 (0.910 - 1.625)	0.186		
Anatomic neoplasm subdivision (Left vs. Right)	516	1.040 (0.772 - 1.401)	0.797		
RPL22L1 (Low vs. High)	530	1.521 (1.132 - 2.044)	0.005	1.542 (1.138 - 2.090)	0.005
